# A Randomized Study of Minimally Invasive Percutaneous Nephrolithotomy (MPCNL) with the aid of a patented suctioning sheath in the treatment of renal calculus complicated by pyonephrosis by one surgery

**DOI:** 10.1186/s12894-016-0184-0

**Published:** 2016-12-08

**Authors:** Jianrong Huang, Leming Song, Donghua Xie, Monong Li, Xiaolin Deng, Min Hu, Zuofeng Peng, Tairong Liu, Chuance Du, Lei Yao, Shengfeng Liu, Shulin Guo, Jiuqing Zhong

**Affiliations:** 1 Department of Urology, The Affiliated Ganzhou Hospital of Nanchang University, 17 Hongqi Avenue, Ganzhou, Jiangxi 341000 China; 2 Department of Urology, Detroit Medical Center, Detroit, MI 48201 USA; 3 Department of Urology, The affiliated Qingdao Municipal Hospital of Medical College of Qingdao University, Qingdao, Shandong 266021 China

**Keywords:** Calculus pyonephrosis, Suctioning lithotripsy and stone clearance sheath, MPCNL

## Abstract

**Background:**

Calculus pyonephrosis is difficult to manage. The aim of this study is to explore the value of a patented suctioning sheath assisted minimally invasive percutaneous nephrolithotomy (MPCNL) in the treatment of calculus pyonephrosis.

**Methods:**

One hundred and eighty two patients with calculus pyonephrosis were randomizely divided into observation group (*n =* 91) and control group (*n* = 91). The control group was treated with MPCNL traditionally using peel-away sheath while the observation group was treated with MPCNL using the patented suctioning sheath.

**Results:**

All the patients in the observation group underwent one stage surgical treatment, 14 patients in the control group underwent first-stage surgery with the rest of the group underwent one stage surgery. The complication rate was 12.1% in the observation group, significantly lower than the rate in the control group which was 51.6%; One surgery stone clearance in the observation group was 96.7% while it was 73.6% in the control group; operative time in the observation group was (54.5 ± 14.5) min, compared to (70.2 ± 11.7) min in the control group; the bleeding amount in the observation group was (126.4 ± 47.2) ml, compared to (321.6 ± 82.5) ml in the control group; the hospitalization duration for the observation group was (6.4 ± 2.3) days, compared to (10.6 ± 3.7) days in the control group. Comparison of the above indicators, the observation group was better than the control group with significant difference (*p* < 0.001 each).

**Conclusions:**

Minimally invasive percutaneous nephrolithotomy with the aid of the patented suctioning sheath in the treatment of calculus pyonephrosis in one surgery is economic, practical, and warrants clinical promotion.

**Trial registration:**

This study was registered with Chinese Clinical Trial Registry on May 18, 2016 (retrospective registration) with a trial registration number of ChiCTR-IOR-16008490.

## Background

**Fig. 1:**
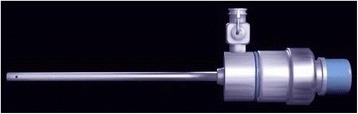
Patented suctioning lithotripsy sheath

Currently MPCNL has become one of the most important means in the treatment of upper urinary calculi. However, inappropriate infusion or poor drainage both can lead to significantly increased renal pelvic pressure, resulting in renal damage at various degrees, liquid reflux or extravasation, infection spread, urosepsis, or infectious shock. Patients undergo surgeries for complicated stones with pyonephrosis are more liable to worsening infection and urosepsis [1]. We designed a patented system with suctioning ability to facilitate minimally invasive PCNL (MPCNL). This patented system was found to be safe and highly efficient in managing renal stones in the previous study [2–4]. However, we did not know the success and complication rates of our MPCNLs in treating renal calculus complicated by pyonephrosis by one surgery. In this study, we performed one-stage MPCNLs using a self-designed and patented suctioning lithotripsy sheath [4] (thereafter referred to as the patent sheath, see Fig. 1) in treating renal and upper ureteral calculi complicated by pyonephrosis in 91 cases to clear stone by one surgery, avoided two stage surgeries including first-stage percutaneous nephrostomy and second-stage stone lithotripsy. We thus reported the outcome as below.

## Methods

### Patients

From March 2011 to April 2013 one hundred eighty two patients with calculus pyonephrosis were admitted and treated at Ganzhou Hospital of Nanchang University, China. Among them 110 were males while 72 were females. The age ranged from 21 to 82 years old with a mean age at 44.1 ± 3.7 years old. There were 98 cases with left renal calculus while 84 cases with right renal calculus. Calculus dimension ranged from 8.8 to 22.5 cm^2^ with a mean dimension at 15.1 ± 6.5 cm^2^. All patients had costovertebral angle (CVA) tenderness by physical exam. Urinalysis revealed white blood cell (WBC) ++ ~ +++. Seventy four patients were found to have Temperature ≥ 37.8 °C. The stone diseases were diagnosed by B ultrasonography, KUB (Kidneys, ureters, and bladder x-ray), or intravenous urography (IVU). Computed tomography (CT) scan was performed for each patient to confirm. Patients with systemic coagulopathy were ruled out from the study. These enrolled patients were randomized into either control group(*n* = 91) or observation group(*n* = 91) in a ratio of 1:1 according to the random number table. As shown in Table 1, comparisons of the two groups on sex, age, stone location, and course of disease revealed no significant difference (*P* > 0.05 each).

**Table 1:** General clinical data comparison(n = 91 each, $\overline{x}$ ±s)

Variable	Observation group	Control group	*P* value
Age (year)	43.5 ± 2.9	44.1 ± 3.2	>0.05
Sex			
Male: Female	53:38	51:40	>0.05
BMI(kg/cm2)	21.6 ± 5.7	22.5 ± 1.1	>0.05
Stone size(mm)	16.7 ± 5.8	15.1 ± 6.3	>0.05
Stone location			>0.05
Renal pelvis and upper segment of ureter	27 (29.7%)	29 (31.9%)	
Upper renal calyx	25 (27.5%)	21 (23.1%)	
Mid renal calyx	9 (9.9%)	7 (7.7%)	
Lower renal calyx	30 (33.0%)	34 (37.4%)	
Renal insufficiency (*n*,%)	8 (8.8%)	11 (12.1%)	>0.05
H/o ESWL (*n*,%)	15 (14.3%)	17 (12.8%)	>0.05
H/o surgery to remove stone (*n*,%)	13 (14.3%)	14 (15.4%)	>0.05
Comorbidities			>0.05
Hypertension	7 (7.7%)	6 (6.6%)	
Diabetes mellitus	4 (4.4%)	6 (6.6%)	
Heart disease	5 (5.5%)	3 (3.3%)	
Stone composition			>0.05
Calcium oxalate	44 (48.4%)	43 (47.3%)	
Calcium phosphate	11 (12.1%)	15 (16.5%)	
Cystine	2 (2.2%)	1 (1.1%)	
Struvite	19 (20.9%)	17 (18.7%)	
Uric acid	7 (7.7%)	5 (5.5%)	
Mixed	8 (8.8%)	10 (11.0%)	
Hydronephrosis			>0.05
Mild	13 (14.3%)	17 (18.7%)	
Moderate	47 (51.6%)	49 (53.8%)	
Severe	31 (34.1%)	25 (27.5%)	

### Surgical procedure

**Fig. 2:**
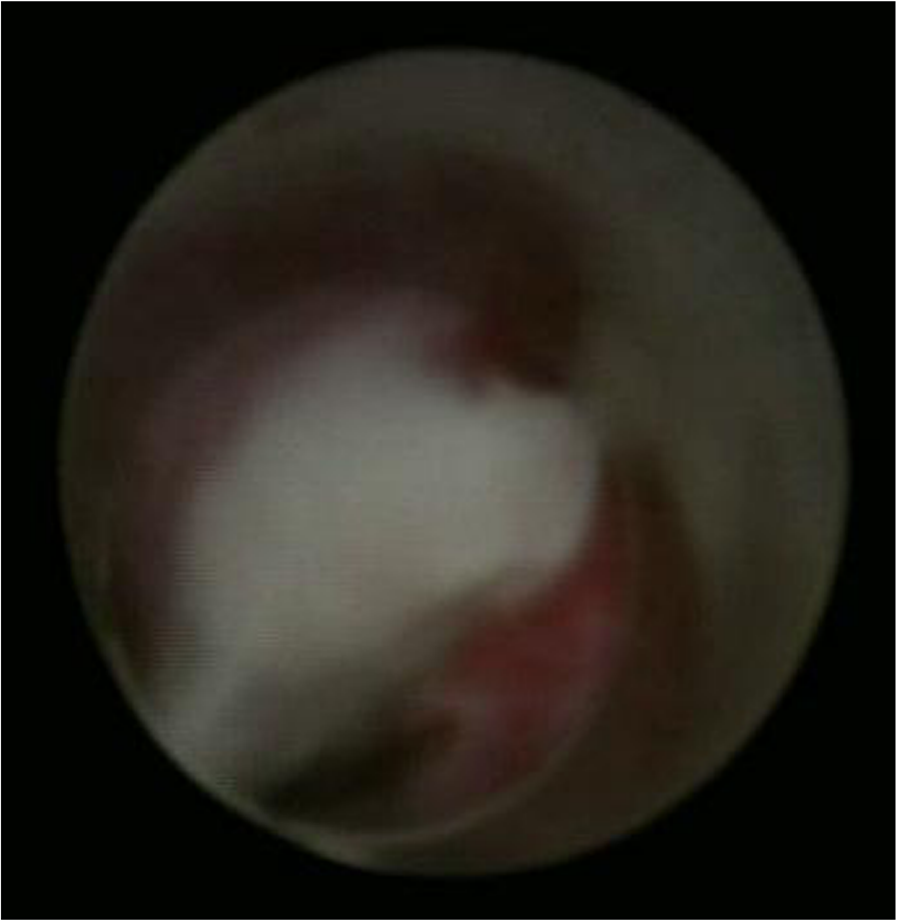
Endoscopic view of renal calculus complicated by pyonephrosis

Surgery was performed under general anesthesia for all 182 patients, all the surgeries were performed by one surgeon. Patients in the observation group underwent patented sheath assisted MPCNL. Patients in the control group were treated using traditional MPCNL without the assistance of the patented sheath but peel-away sheath. Patients in both groups were administered antibiotic pre and post surgery. The patient was first placed in a lithotomy position. A 5 F ureteral catheter was then inserted retrogradely into the renal pelvis through cystoscopy or ureteroscopy, and continuous infusion of saline was used to produce artificial hydronephrosis. After this, a Foley catheter was inserted; the patient was then changed to the prone position. Ultrasonography-guided percutaneous punctures were made with an 18-gauge coaxial needle into the targeted calyx. The puncture point was in the 11th intercostal space or the 12 th subcostal margin, between the posterior axillary line and scapula line. The puncture was judged successful if there was urine overflow or if it touched a stone. Zebra guidewire was inserted and fixed. The puncture needle was then taken out. After a 0.5–0.7 cm skin incision, the dilation of the percutaneous tract was performed serially over the guidewire with a fascial dilator from 8 F to 16 F. A 16 F patented sheath was then placed at the percutaneous access port and was connected to a vacuum aspiration machine. The pressure was maintained at 0.01–0.02 MPa. Subsequently, a small diameter nephroscope was inserted through the sheath. Initially, pus or purulent bolts were sucked out using the vacuum suctioning device (See Fig. 2). A holmium laser was then used to break the stones, and a vacuum suctioning device was used to clear gravel through the patented sheath until complete clearance. We then inspected renal pelvis and each renal calyx carefully. After finding that all stones were cleared, we indwelled a 6 F ureteral stent and a 16 F nephrostomy tube which will be removed 4–5 days after surgery. Occurrence of renal pelvic perforation was investigated at the end of the procedure via nephrostogram.

For any patient in the two groups with the following intraoperative occurrences, the surgery was converted to a simple nephrostomy: (1) body temperature lower than 36 °C or higher than 38 °C; (2) heart rate higher than 90 beats per minute; (3) respiratory rate greater than 20 breaths per minute or PaCO2 less than 32 mmHg [5]; (4) excessive bleeding;(5) suctioning channel was blocked due to thick pus.

### Observation index

Perioperatively, vital signs were closely monitored. Also, the complete blood count, serum electrolytes, BUN/creatinine, and calcitonin were checked. Patients were evaluated on postoperative day 30 by KUB assess stone-free status. For patients with radiolucent or residual stones, CT scan was performed. Successful stone clearance was defined as no residual stone or residual stone size < 4 mm. Patients in the two groups were compared for operation time, bleeding amount, stone clearance rate, complication rate, and average days of hospitalization.

### Statistical analysis

We used Excel for data input. SPSSll.5 statistical software package was used for data analysis. The measurement data were represented as mean ± standard deviation ($\overline{x}$ ±s) and analyzed using *t* test. Count data was analyzed using *χ* ^2^ test. *P* < 0.05, with statistically significant difference.

## Results

**Fig. 3:**
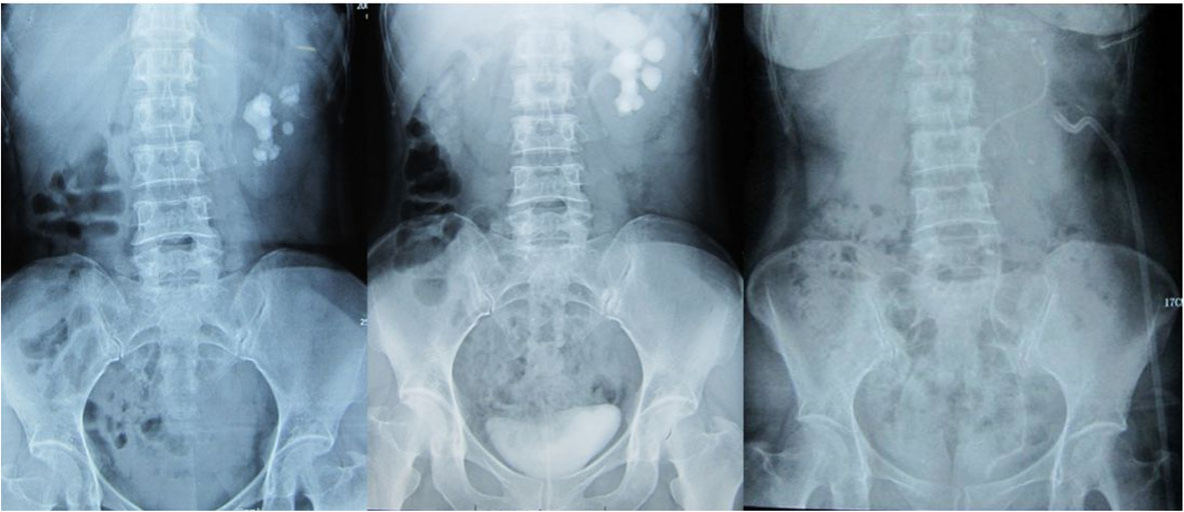
Left: Preoperative KUB for a patient with staghorn renal calculus complicated by pyonephrosis; Middle: Preoperative IVU for a patient with staghorn renal calculus complicated by pyonephrosis; Right: Post-MPCNL KUB for a patient with staghorn renal calculus complicated by pyonephrosis, with the aid of the patented suctioning sheath

All patients in the observation group were treated with patented sheath assisted MPCNL successfully by one surgery without major complication including surrounding organ injury, pleural effusion, or major bleeding. There were 75 cases stones were cleared through one percutaneous tract, 15 cases stones was cleared through two percutaneous tracts, and two cases stones were cleared through three percutaneous tracts. There were ten cases with temperature ≥ 38.5 °C on postoperative day 2. There was one case in this group with complication of renal pelvic perforation. For the patients in the control group, 14 cases underwent first-stage percutaneous nephrostomy due to the difficulty in doing lithotripsy in the first surgery, while the rest of this group underwent one stage MPCNL. There were 15 cases with bleeding ≥ 800 ml and were transfused. There were 25 cases with temperature ≥ 38.5 °C on postoperative day 2. There were seven cases in this group with complication of renal pelvic perforation. In the observation group each index is better than the control group with significant difference (*P* < 0.001 each, see Table 2). Body temperatures returned to normal for those patients with postoperative fever after strengthening anti-infection treatment without the occurrence of sepsis or pyemia. After a follow up of 3 months, serum creatinine (Scr) and blood urea nitrogen (BUN) in 74 cases that had elevated preoperative Scr and BUN preoperatively were reduced to varying degrees (See Table 2 and Fig. 3).

**Table 2:** Operative outcome comparison between two groups($\overline{x}$ ±s)

Variables	Observation group	Control group	*P* value
Operation time (min)	54.5 ± 14.5	70.2 ± 11.7	<0.001
Bleeding amount (ml)	126.4 ± 47.2	321.6 ± 82.5	<0.001
Stone-free rates	96.7% (88)	73.6% (67)	<0.001
Incidence of complication			
Fever ≥ 38.5 °C	10	25	<0.001
Bleeding amount ≥ 800 ml	0	15	<0.001
Renal pelvic perforation	1	7	<0.001
Days for hospitalization	6.4 ± 2.3	10.6 ± 3.7	<0.001

## Discussion

Upper ureter or renal stones often were treated with MPCNL and most patients can achieve the purpose of cure. Due to the pressure limit of renal parenchymal reflux is 30 mmHg [6], when the MPCNL is performed under high pressure perfusion it is easy to cause the intrapelvic pressure over 30 mmHg, and the operation manipulation, the integrity of the pelvic wall epithelium could be injured and thereafter leads to direct exposure of venous and lymphatic system followed by renal parenchyma reflux [7]; When the stones and infection occur at the same time, tissue edema and congestion are more likely to cause pelvic fluid absorption. A large amount of short-term liquid absorption can cause the perfusion fluid syndrome, and when the bacteria and its toxin reflux into the blood, complications like bacteremia, sepsis, or postoperative fever occur [8]. MPCNL surgery complication is related to the amount of liquid absorption. It is a positive correlation between the integrity of the epithelial cells, renal pelvic pressure, and the operation time [6, 9]. Performing MPCNL through irrigation can cause stones shift. When the collection system is connected to the renal abscess pus, due to the blurring of vision, it is not easy to find the pelvic outlet so the surgeon is often forced to abandon stone lithotripsy in which circumstance usually a percutaneous nephrostomy is performed as a first-stage surgery. In the current study, incidence rate of surgical complications in the control group was 51.6%% with 14 cases needing two staged operation. Therefore, how to improve success rate of surgery and reduce postoperative complications for calculus pyonephrosis is always a challenge in the field of Urology [10].

According to a previous report, when the intra-pelvic pressure is below 20 mmHg, it is feasible to use the EMS LithoClast master for one-phase PCNL of the relatively symptomatically stable patients with calculous pyonephrosis. Nevertheless, this surgical procedure has always carried high risks and its advantages and disadvantages should be validated by further studies of larger sample sizes [11, 12]. Patented sheath connected during surgery remains 0.01–0.02 MPa negative pressure suction to keep renal pelvis in a negative pressure state, so that the discharge of perfusion fluid and pus went smoothly, avoided lavage, bacteria, toxins reflux and spread to surrounding tissues, reduced fever infection complications after surgery. In the current study, only 10 patients in the observation group were found to have temperature ≥ 38.5 °C, significantly lower than those in the control group; Negative pressure adsorption can remove the effect of blood clots and floc, the vision can be more clear. With negative pressure “adsorption” effect on the gravel, small stones and pus hidden in the calyces can be automatically removed by suction. Due to a big discharge cavity of the sheath which is not easy to be blocked, there is no need of lithotomy forceps or stone basket, without the need for repeated importing a ureteroscope to flush, either. We are thus able to improve the efficacy of lithotripsy and stone clearance, shorten operation time, and reduce the operation complications such as bleeding [3]. In this study, all patients in the observation group were successfully treated with the MPCNL by one surgery with a stone clearance rate of 96.7%. There were no adjacent organ injury and major bleeding, compared with the control group, with significant difference. With improved success rate of surgery and reduced rate of complication, the hospitalization time was shortened and the cost of hospitalization was reduced. Of course, surgeons may have been more aggressive using the patented sheath and less aggressive with the peel-away sheath due to the aims of this study, therefore getting higher stone-free rates in patients in the observation arm. Other limitation of this study is that we did not measure intrarenal pelvic pressure for every patient during the surgery, even though we have had previous research data revealing that the patented sheath could reduce intrarenal pressure.

## Conclusion

In summary, through analysis and comparison the observation group was better than the control group reflected by multiple clinical indexes. Managing calculus pyonephrosis using patented sheath assisted MPCNL is safe, economic with fewer complications.

## Abbreviations

BUN: Blood urea nitrogen
CT: Computed tomography
CVA: Costovertebral angle
IVU: Intravenous urography
KUB: Kidneys, ureters, and bladder x-ray
MPa: Megapascal
MPCNL: Minimally invasive percutaneous nephrolithotomy
PaCO2: Partial pressure of carbon dioxide in arterial blood
PCNL: Percutaneous nephrolithotomy
Scr: Serum creatinine (Scr)
WBC: White blood cell
